# The value of epidemiology in headache

**DOI:** 10.1186/1129-2377-16-S1-A43

**Published:** 2015-09-28

**Authors:** Ettore Beghi

**Affiliations:** Department of Neuroscience, IRCCS Institute for Pharmacological Research Mario Negri, Milan, Italy

Headache is the commonest neurological condition worldwide. According to the Global Burden of Disease 2010, tension-type headache is the second most common clinical condition (prevalence, 20.8%) preceded only by dental caries and followed by migraine (prevalence, 14.7%)[1]. Globally, migraine is the leading cause of disability-adjusted life years (DALY) and years living with disability (YLD). This information was obtained from epidemiological studies in developed and in developing countries where well-defined populations were investigated in search of people suffering from headache. Epidemiological studies estimate the frequency, spectrum and burden of a disease in a population. Headache, in this regard, has peculiar aspects that cannot be fully explored when studying referral patients (i.e., individuals seen in hospital, headache centers and other outpatient services) as the majority of patients do not have access to hospital facilities and several individuals do not even ask for medical consultation. However, in light of the frequency of headache in the population, the disease has a high impact on the society in terms of functional disability and indirect costs[2].

As several epidemiological studies are based on telephone interviews, the full spectrum of the disease can be easily captured on a population basis. Epidemiological studies are also instrumental when addressing the role of personal and environmental risk factors and their association with headache (migraine) types[3]. However, the representativeness of the study population is a pre-requisite for the external validity of the results. In addition, in the absence of valid and reliable biomarkers, the diagnosis of headache still relies mostly on clinical findings[4]. In this regard, epidemiology largely contributes to the identification of symptoms and signs most likely to differentiate the main diagnostic categories of the disease.
